# Nulligravida with Large Uterine Leiomyoma: A Case Report

**DOI:** 10.31729/jnma.7333

**Published:** 2022-06-30

**Authors:** Sujata Maharjan, Meena Thapa, Manoj Pokhrei

**Affiliations:** 1 Department of Obstetrics and Gynaecology, Kathmandu Medical College and Teaching Hospital, Sinamangal, Kathmandu, Nepal

**Keywords:** *case reports*, *gonadotropin-releasing hormone*, *hysterectomy*, *leiomyoma*

## Abstract

Uterine leiomyoma is the most common benign tumour of the female reproductive tract originating from the uterine smooth muscle causing morbidity and impairing their quality of life. It is common among women in the age group 30 to 50 years of age. Women are usually asymptomatic or may present with various symptoms such as abnormal uterine bleeding, pelvic pain, dysmenorrhea, and change in bowel and bladder habits due to pressure symptoms. It is one of the leading causes of hysterectomy. Women with uterine leiomyoma can be managed medically and surgically. Gonadotropin-releasing hormone analogue is one of the modalities used preoperatively to reduce the size of large uterine fibroid. We present the case report of a 36-year-old nulligravida who underwent total abdominal hysterectomy with bilateral salpingectomy for large uterine leiomyoma weighing 5.61 kg without compression symptoms. She received a gonadotropin-releasing hormone agonist (injection leuprolide) preoperatively for reduction of the size of uterine myoma.

## INTRODUCTION

Uterine leiomyomas are the most common uterine neoplasms originating from uterine smooth muscle cells (myometrium).^1^ Usually asymptomatic, but they can present with abnormal uterine bleeding, pressure symptoms to bowel and bladder and back pain. Treatment of uterine leiomyomas depends on the patient's age and family planning goals, as well as tumour size and symptomatology. Fibroids account for 39% of all hysterectomies annually.^2^ Recently emerging modalities for the management of fibroid include uterine artery embolization, high intensity focused ultrasound and Magnetic Resonance Imaging (MRI) guided focused ultrasound. Medical management used for short term therapy includes Gonadotropin-releasing Hormone (GnRH) agonists, Selective Estrogen Receptor Modulators (SERMs), antiprogestins and aromatase inhibitors.^3^

## CASE REPORT

A 36-year-old, nulligravida presented to the gynaecological Out Patient Department (OPD) with gradual abdominal distention for 3 years which was mistaken as abdominal obesity by the patient. There was no history of abdominal pain, abdominal discomfort, difficulty in micturition, difficulty in defecation, difficulty in breathing, swelling of lower limbs, engorgement of veins, nausea, vomiting, weight loss, or anorexia. However, she had occasional dyspareunia. She was married for 19 years and was nulligravida who never planned for conception and denied workup for subfertility. She had menarche at 14 years of age with a regular menstrual cycle and four period days soaking three pads per day. She denied a history of dysmenorrhea, heavy menstrual bleeding, intermenstrual bleeding and postcoital bleeding. She denied the use of contraceptives. There was no significant personal or family history.

A preliminary physical examination was performed indicating good general condition and no evidence of pallor or pedal oedema. Her Body Mass Index (BMI) was 34.8 kg/m^2^. Per abdomen examination revealed abdomen distended corresponding to 36 weeks gravid uterus size which was firm with smooth surface, non-tender, nonmobile with flank fullness on both sides. There were no hernias or abdominal varices seen.

Her laboratory analysis showed blood haemoglobin of 15 gm/dl with other preoperative investigations including renal function test, liver function test, Cancer Antigen 125 (CA-125), and thyroid function test within normal limits. Pregnancy was excluded. Her transabdominal ultrasonography revealed a large abdominopelvic mass of 45x18 cm with increased vascularity without separate visualization of the uterus. A well-defined cystic lesion measuring 73x39 mm was noted in the left adnexa likely an ovarian cyst. She was counselled for Contrast-enhanced Computed Tomography (CECT) of the abdomen and pelvis which revealed a large homogeneous soft tissue density lesion measuring 25x24x19 cm in the abdominopelvic cavity continuous with the fundus of the uterus suggesting an origin from the uterus likely uterine fibroid. A lobulated cystic lesion in the bilateral adnexa with few septa is likely bilateral hydrosalpinx with mild pelvic ascites.

**Figure 1:**
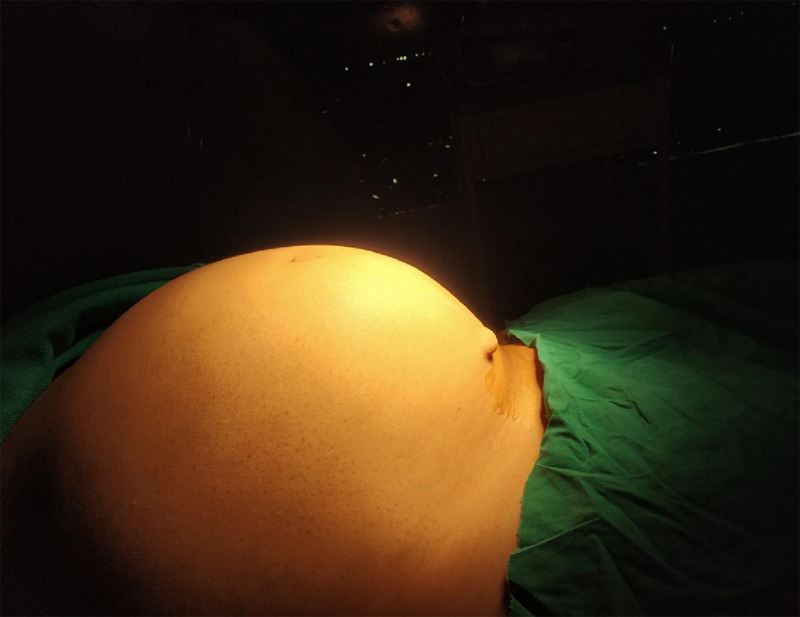
Abdomen distension up to 32 weeks gravid uterus size.

The patient was counselled for GnRH analogues preoperatively for fibroid size reduction. She received Injection of leuprolide 3.75 mg subcutaneously 4 weekly for 3 months. Per abdomen, mass decreased to 32 weeks gravid uterus size (Figure 1).

**Figure 2:**
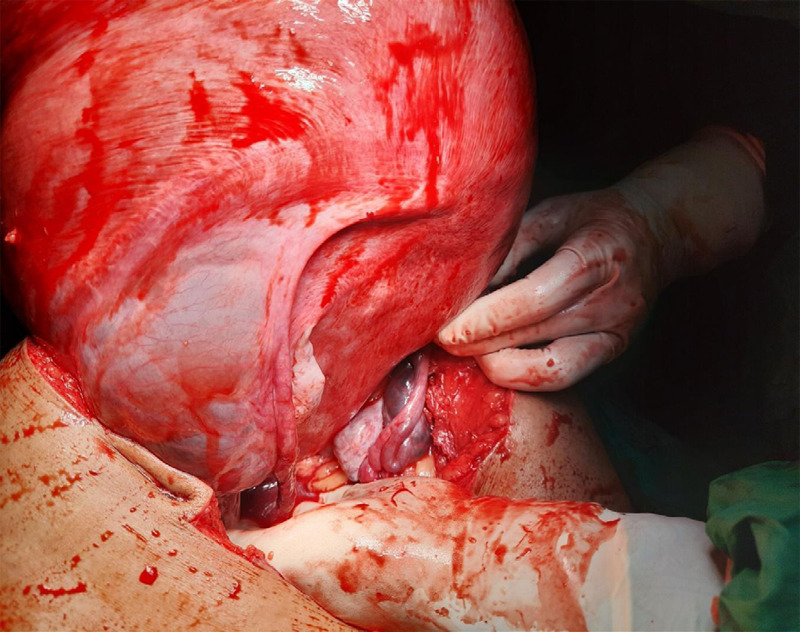
Cystic collection over mesosalpinx with bilateral normal fallopian tubes and ovaries.

She was planning for a total abdominal hysterectomy. Preoperative investigations were reviewed. She underwent laparotomy after proper counselling and written informed consent. Laparotomy was done under general anaesthesia and an epidural catheter was used for postoperative pain management. A vertical midline incision was made extending from midway between the xiphisternum to the umbilicus to pubic symphysis. Intraoperatively, an enlarged uterus with intramural fibroid filled the entire abdominal cavity with 100 ml of ascites. No adhesions were noted. A multilobulated cyst was present over bilateral mesosalpinx containing 200 ml of straw-coloured fluid (Figure 2).

**Figure 3:**
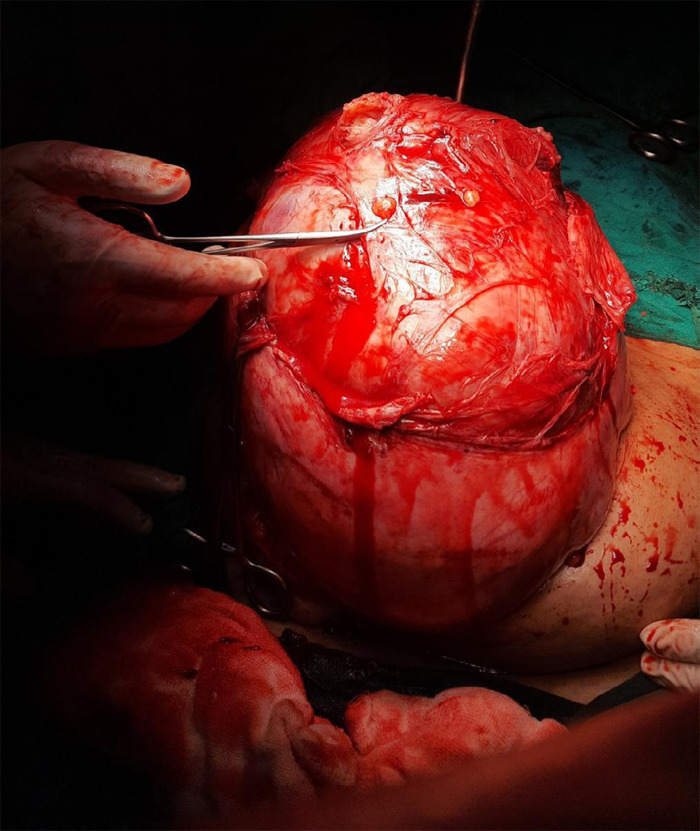
Intraoperative image of the exteriorized uterus with fibroid.

Both fallopian tubes and ovaries were normal. A transverse incision was given over the fundus of the anterior uterine wall until myoma was visible. The uterus was exteriorized with help of a myoma screw over myoma which extended the uterus longitudinally decreasing the transverse diameter of the uterus (Figure 3).

**Figure 4:**
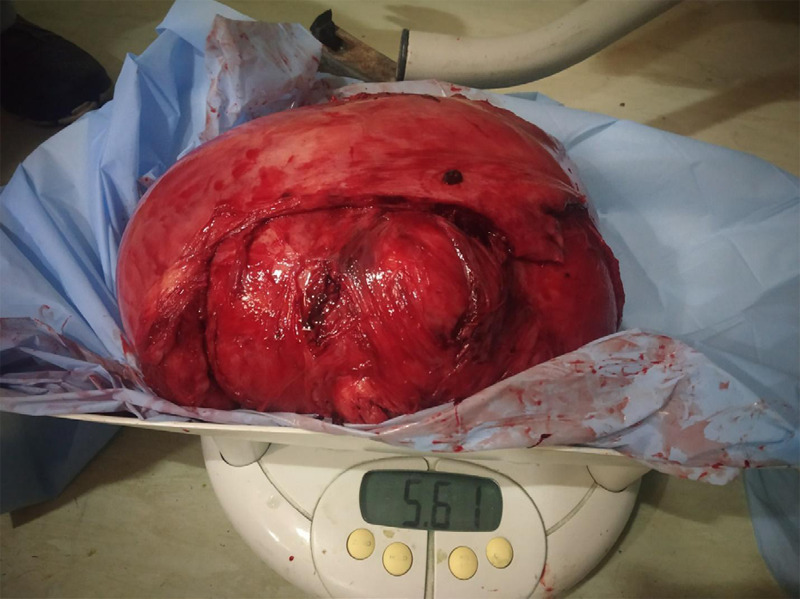
Uterus along with fibroid weighing 5.61 kg.

Total abdominal hysterectomy with bilateral salpingectomy was performed with an estimated blood loss of 1500 ml. Intraoperatively, one unit of whole blood was transfused. The abdomen was closed in layers. The gross specimen was weighing 5.61 kg, uterus was measuring 30x25x20 cm with a smooth surface, uniformly enlarged (Figure 4).

On cut section, revealed a single encapsulated whitish intramural myoma of 28x20x12.5 cm with fibroelastic tissue arranged in a whorled pattern. No haemorrhage and necrosis were seen. The specimen was sent for histopathological examination which confirmed the diagnosis of uterine leiomyoma without degenerative changes.

She was shifted to a postoperative ward where a second and third unit whole blood transfusion was done. Postoperatively her vitals were stable. Her postoperative haemoglobin after three units of blood transfusion was 10.6 gm/dl injection antibiotic prophylaxis was given as per hospital protocol. She received thromboprophylaxis for 5 days. She was called for follow up on her tenth postoperative day for suture removal and was uneventful.

## DISCUSSION

Uterine leiomyomas are the most common benign tumour of the female reproductive tract among the age group 30 to 50 years. Associated epidemiological factors for the development of uterine fibroids include reproductive factors, age, race, heritage, sex hormones, obesity, lifestyle, environmental influences and infection. Most women with uterine leiomyomas are asymptomatic. While most common symptoms are pelvic pain, abnormal uterine bleeding, and pressure symptoms like voiding difficulty, fatigue, loss of appetite, dyspnea and chronic constipation. Myomectomy or hysterectomy may be done according to the woman's choice and her desire for future childbearing in premenopausal women.^4^

The largest uterine fibroid ever reported to date weighed 63.3 kg which was removed postmortem in 1888. While the largest tumour ever removed from a patient who survived a procedure weighed 45.4 kg.^5^ A major university in Texas reported a 33-year-old woman with 11.6 kg of uterine fibroid presents with anaemia and abdominal distension who underwent total abdominal hysterectomy with bilateral salpingo-oophorectomy.^6^ A large fibroid was reported in India, 38 years old premenopausal woman with giant uterine leiomyoma weighing 15.2 kg who underwent a total abdominal hysterectomy.^7^ Our case was 36 years old asymptomatic woman who underwent a total hysterectomy with bilateral salpingectomy with an uneventful postoperative period.

Currently, GnRH agonists are used primarily as preoperative therapy, and a Cochrane systematic review has demonstrated how their use can improve both preoperative and postoperative haemoglobin levels, reduce operative time, and shorten the duration of hospital stay.^8^ These outcomes are achieved by the action of GnRH agonists in decreasing menstrual bleeding and reducing fibroid volume (and therefore uterine volume) by approximately 50%.^9^

In a study done in Italy, among 36 patients who were planned for myomectomy, 20 patients received longterm GnRH analogue administration, six-monthly depot injections of leuprolide acetate (LA), while 16 patients were treated with 2 monthly LA injections before the surgery. The uterine volume decrease was statistically significant after two LA injections in both groups while the decrease observed between two and six LA injections was not significant.^10^ In a clinical trial done in Germany, among 114 women, a mean reduction of the uterine volume of about 67% was observed, in conjunction with shrinkage of the myoma in 92.1 % of cases (mean decrease of 56% of the fibroids) with a large inter-individual difference. Maximal diminution of uterine and fibroid size had been nearly completely reached within the first 12 weeks of therapy.^11^ We used an injection of leuprolide acetate 4 weekly three doses prior to surgical intervention. There was a reduction in the size of the uterus from 36 weeks gravid uterus to 32 weeks gravid uterus size but the ultrasonographic volume of fibroid size was not determined.

Leiomyoma greater than the reported case could be faced in developing countries like Nepal where people are unable to access available health facilities for a regular health checkup. Surgical intervention is a great challenge to gynaecologists in such cases due to associated postoperative complications. With the use of GnRH analogues, diminution in the size of leiomyoma could be an aid during surgical intervention.
